# An Ergonomic Risk Assessment of Ophthalmology Residents Using the Rapid Entire Body Assessment (REBA) Scale

**DOI:** 10.7759/cureus.53698

**Published:** 2024-02-06

**Authors:** Avery K Morrison, Savannah Kumar, Abha Amin, Matthew Urban, Ben Kleinman

**Affiliations:** 1 Ophthalmology, New York Medical College, Valhalla, USA; 2 Ophthalmology, Northwell Health, Great Neck, USA; 3 Ophthalmology, New York Medical College/Westchester Medical Center, Valhalla, USA

**Keywords:** slit lamp examination, posture, reba, resident, ergonomics

## Abstract

Background: The healthcare industry has one of the highest rates of work-related injuries. Ophthalmologists are at particularly high risk for developing musculoskeletal disorders (MSDs), which are often the result of repetitive movements, such as performing slit lamp examinations.

Previous studies used the Rapid Entire Body Assessment (REBA) scale to determine the ergonomic risk of a particular task.^ ^Higher REBA scores correlate with increased risk of injury, which ranges from negligible risk (1) to very high risk (11+).

Objective: Given the long-term implications of repetitive examinations by ophthalmologists, this study aims to describe the average ergonomic risk posed to residents using the REBA scale.

Methods: This descriptive case study assessed four ophthalmology residents performing slit lamp examinations between September 2022 and February 2023. Photographs were taken of residents performing slit lamp examinations. Total REBA scores, Score A, Score B, and the REBA scores for each participant were calculated and compared.

Results: The average REBA score across all participants was 4.59 (SD±0.89). The highest score was 7.00 and the lowest was 3.00. The average Score A, representing posture for the head, leg, and trunk, was 3.54 (SD±0.74) and the average Score B, representing posture for the upper arm, lower arm, and wrist, was 3.18 (SD±1.22).

Conclusion: Our study found that residents are at increased risk for developing MSDs. Furthermore, variation in REBA scores between residents indicates that not all residents are at equal ergonomic risk. This highlights an opportunity for ophthalmology residency programs to implement ergonomics training into their curriculum.

## Introduction

The healthcare industry has an elevated frequency of work-related injuries, surpassing those observed in any other private sector, including manufacturing, with over 800,000 cases reported in 2020 [1]. In particular, surgical specialties have been shown to have an increased risk for work-related overuse injuries, such as carpal tunnel syndrome, neck tension syndrome, cervical spine disease, lumbar degenerative disease, shoulder and wrist tendonitis, rotator cuff injury, and trigger finger [1,2]. Surgeons often hold awkward postures in “bent, extended, or flexed positions rather than straight or neutral positions,” which can lead to musculoskeletal (MSK) injury when performed repetitively [1]. In a meta-analysis including 5,152 surgeon surveys among all specialties, 68% reported generalized pain, 71% reported fatigue, 37% reported numbness, and 45% reported stiffness [2].

Ophthalmologists commonly complain of neck and back pain from overuse injuries [3]. These injuries are often the result of repetitive movements performed during slit lamp examinations, indirect ophthalmoscopy, and surgical procedures [2,3]. For example, during slit lamp examinations, the head, neck, and torso should ideally be aligned vertically in neutral posture. However, ophthalmologists often lean toward the slit lamp, moving the neck out of alignment and into extension repeatedly [4]. As a result of their injuries, ophthalmologists report turning to pain medication, reducing their hours, and retiring early [3].

To assess the ergonomic risk faced by medical professionals, other studies, such as that by Raman et al., have employed the Rapid Entire Body Assessment (REBA) scale. The REBA scale has been used to quantify and describe the chairside ergonomic risk of particular procedures and examinations using digital photographs [5]. Use of the REBA scale as a posture evaluation tool has increased recently because it is sensitive to musculoskeletal disorder (MSD) risk across a variety of tasks [5,6]. It was developed by a team of ergonomists, physiotherapists, occupational therapists, and nurses to account for dynamic and static postural load forces for a specific task. Additionally, it accounts for how the person interacts with a machine during a task, such as handgrip and machine safety, with the Coupling Score [7].

The REBA scale has been used by others, such as Aaron et al., to assess ergonomic risk across multiple surgical specialties [8]. This study found ophthalmology had the lowest risk of MSDs across cardiology, general surgery, interventional radiology, neurosurgery, obstetrics and gynecology, orthopedics, otolaryngology, plastics and vascular surgery [8]. However, compared to this study, which focuses on REBA scores for slit lamp examinations, Aaron et al. focused on intraoperative REBA scores [8].

An additional study in Iran used the REBA score to evaluate the effectiveness of ergonomic training on work-related injuries for 74 operating room nurses [9]. After an initial REBA score was assigned, participants were either placed in an intervention or control group [9]. The intervention group went through an ergonomics educational program and the control group did not get training [9]. At the end of three months, those in the intervention group saw a reduction in the risk of MSDs (p<0.05) [9]. Ideally, assessing ergonomics in ophthalmology using the REBA scale will result in a similar practice change to improve MSD risk.

Considering the adverse and enduring MSK effects of conducting repetitive examinations, this study seeks to assess the average ergonomic risk associated with performing slit lamp exams utilizing the REBA scale [1]. This study focused specifically on ophthalmology residents in order to uncover whether recently trained ophthalmologists are already at risk of developing MSDs.

## Materials and methods

This IRB-approved, descriptive case study assessed four ophthalmology residents performing slit lamp examinations (n=22, 100% response rate) in an outpatient clinic setting between September 2022 through February 2023. Physicians from other specialties, from other institutions, and those at or above the level of attending were not included in this study. This study excluded individuals at or above the attending level to investigate whether recently trained ophthalmologists face a higher risk of developing MSDs compared to those in later stages of their training who have established ergonomic habits. The study was reviewed by the New York Medical College Institutional Review Board prior to commencement and was deemed as a quality assurance and quality improvement study.

**Figure 1:**
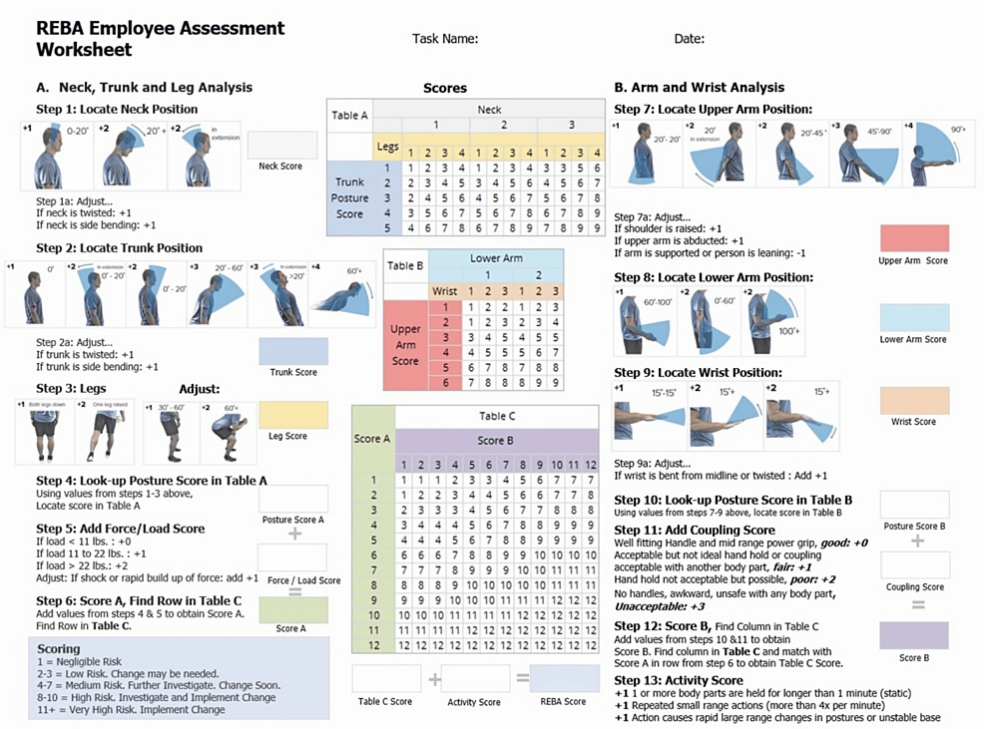
REBA scoring worksheet Source: Reference [7] REBA: Rapid Entire Body Assessment

The REBA scale was used to evaluate ergonomic injury risk after obtaining verbal and written consent from both the patient and resident [9]. The REBA scale is a method used to assess forced postures, as shown in Figure 1. The REBA scores range from 1 to 15. Increases in REBA scores correlate with increased risk for ergonomic injury for a specific task, which include negligible risk (1), low risk (2-3), medium risk (4-7), high risk (8-10), and very high risk (11 and above).

Photographs were taken of the residents performing slit lamp examinations from the anterior, posterior, and side views. These photographs were used to analyze posture using the REBA scale. A grader determined the REBA score through direct observation of the photographs in comparison to the REBA worksheet. Multiple photographs were taken throughout a slit lamp exam to determine an average REBA score across different steps of the slit lamp exam. The same resident was photographed across an average of 5.5 different slit lamp exams. The number of slit lamp exams observed varied between residents. The final REBA score for each patient encounter and the average for each resident were calculated by one grader and stored in an Excel document. Furthermore, for each resident and across all residents, the average Score A and Score B were calculated and reported. 

**Figure 2:**
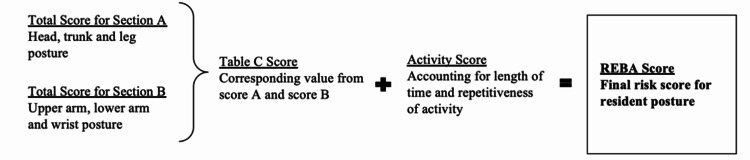
Steps in a REBA score calculation REBA: Rapid Entire Body Assessment

Figure 2 shows the process of calculating a REBA score. First, the total score for section A was calculated, after the observer determined the positioning, torsion, bending, and force load of the head, trunk, and leg in a photograph. Then a similar score was calculated for section B, after the observer determined the positioning, bending, abduction, leaning, and raised shoulders for the upper arm, lower arm, and wrist in a photograph. Scores A and B were then used to find a corresponding Table C score. Lastly, the activity score, which accounts for the length of time in a position and the repetitiveness of the activity, was combined with the Table C score to calculate the REBA score. All residents were assigned an activity score of +1 because the average slit lamp examination requires residents to hold their arm in abduction and flexion for over one minute, which equates to an activity score of one.

## Results

The average REBA score across all participants was 4.59 (SD±0.89, 95% CI [4.218 - 4.962]) (Table 1). The average Score A was 3.54 (SD±0.74, 95% CI [3.231 - 3.849]), representing posture for the head, leg, and trunk; and the average Score B, representing posture for the upper arm, lower arm, and wrist was 3.18 (SD±1.22, 95% CI [2.670 - 3.690]) (Table 1). There was greater variability in the upper extremity posture across ophthalmology residents, indicated by Score B. The highest REBA score was 7.00 and the lowest was 3.00 (Table 1). A complete sample calculation is shown in Table 2.

**Table 1:** REBA score, Score A for lower extremity, and Score B for upper extremity analysis by participants REBA: Rapid Entire Body Assessment

Individual Resident REBA Score Calculations	Resident 1	Resident 2	Resident 3	Resident 4	Total REBA Score
# of slit lamp exams	6	6	8	2	-
Mean	4.17	4.63	5.33	3.50	4.59
Median	4.00	5.00	5.00	3.50	5.00
Minimum	4.00	3.00	5.00	3.00	3.00
Maximum	5.00	6.00	7.00	4.00	7.00
Mean difference	0.42	0.03	0.74	-1.1	-
Score A mean	3.00	3.50	4.33	3.00	3.54
Score B mean	3.33	3.38	3.17	2.00	3.18

**Table 2:** Sample REBA score calculation for residents 1 and 3 REBA: Rapid Entire Body Assessment

REBA Score Calculation Breakdown	Resident 1	Resident 3
Score A for neck, trunk and leg posture	3.00	5.00
Score B for upper arm, lower arm and wrist posture	2.00	5.00
Table C score	3.00	6.00
Final REBA Score	4.00	7.00

After the mean REBA score for each resident was calculated, variation in REBA scores between residents was determined. The one-way ANOVA found significant variation between residents' REBA scores [F(2,15)=4.78, p=0.024806]. This indicates that not all residents are at equal ergonomic risk while performing the slit lamp exam (p<0.05). Importantly, it also shows a potential opportunity for improved education around correct slit lamp posture to minimize REBA scores to the lowest possible value.

## Discussion

Our findings illustrate that ophthalmology residents at this institution are at increased risk for developing MSDs compared to a normal healthy individual. The average REBA score across all ophthalmology residents was 4.59 (SD±0.89, 95% CI [4.218 - 4.962]. A REBA score of 4 to 7 is considered medium risk, which indicates a need for posture change to prevent MSDs. It is alarming that ophthalmology residents are already at risk for developing MSDs this early in their careers. This highlights the need for formal ergonomics training during residency to prevent slit lamp examination-related injuries in the future.

There was also greater variability in Score B across the ophthalmology residents, which correlates with the risk for upper extremity injury as a result of the slit lamp examination. This indicates that some ophthalmology residents are at higher risk of upper extremity injury compared to their colleagues. Often ophthalmologists later in their careers face injuries of the cervical spine and carpal tunnel [3]. Therefore, posture training should focus on mitigating cervical spine and upper extremity strain during the slit lamp examination for ophthalmology residents at higher risk.

This study also found that mean REBA scores varied significantly between residents, indicating that residents face different risks for MSDs early in their careers. Some residents, such as females or those of shorter stature, may be at higher risk of MSDs because of poorly designed ophthalmic examination rooms. Office chairs used during the slit lamp exam with inadequate back support often contribute to the increased risk of MSD for examiners of short stature [10-12]. Other studies, such as that by Sutton et al., have demonstrated that female surgeons are at increased risk of MSK injuries [10]. The increased risk can be attributed to the traditional design of operating rooms and instruments, which have historically favored surgeons with greater height, larger hands, and increased strength [9].

Furthermore, within a training setting, residents may be hesitant to modify slit lamp oculars and chair positions following an attending physician to avoid inconvenience or due to their inexperience [10]. This situation highlights the need to educate medical practitioners about the importance of customizing in-office exam ergonomics to mitigate the risk of long-term MSDs and reduce pain. A 2022 study found that increased awareness and education around ergonomics significantly helped reduce pain and improve function among ophthalmologists [13].

Ideally, formal ergonomics training should be incorporated into resident training to improve resident’s posture during slit lamp exams, indirect ophthalmoscopy, and ophthalmic surgeries. A prior 2019 Canadian study created an educational program for ophthalmologists on the proper ergonomics of the slit lamp examination [10]. They found improvement in injury risk scores after completion [10]. This indicates ophthalmology residents in the United States may also benefit from a formal ergonomics training in their residency programs. Another study showed that occupational therapists are successful in assessing residents' posture using the REBA scale [14]. Partnering with occupational therapists to optimize residents' posture represents another potential training solution in ophthalmology residency. Additionally, another study implemented Upright Go, a postural training device, during ophthalmology residency training [15]. They saw a 27.3% increase in time spent in the proper posture after the completion of the training period [15]. All of these training programs represent cost-effective solutions that can be implemented easily into ophthalmology residency schedules and ultimately improve residents’ career longevity and decrease their risk of developing MSDs.

The American Academy of Ophthalmology also identified ergonomics as an issue ophthalmologists face and AAO established a task force to address the ergonomic risk factors leading to MSDs [4]. This task force offers online courses for proper ergonomics in the workplace for ophthalmologists, which could improve resident posture as well if incorporated into their training [4]. Other specialties outside of ophthalmology have also looked into formal ergonomics programs, such as gynecology, general surgery, and medicine, as ergonomics has become a more openly discussed topic [16-18]. In fact, publications about ergonomics have increased 3-fold from 1972 to 2020 with 1972 seeing nearly 25 citations and 2020 seeing almost 20,000 [19]. A focus of the healthcare industry is improving ergonomics after physicians and healthcare administrators noticed the impact on career longevity and quality of life [20]. This offers a unique opportunity for the field of ophthalmology to embrace the ongoing trend of integrating new ergonomic curriculum into residency programs.

Strengths and limitations

To our knowledge, this case study is the first of its kind investigating the ergonomic risk factors faced by ophthalmology residents using the REBA scale. To achieve this, we analyzed photographs of resident’s postures held while performing slit lamp examinations using the REBA scale. Importantly, the REBA scale successfully identified ergonomic risks faced by residents early in their training, which presents an opportunity to improve the longevity and quality of life of ophthalmologists across the field with earlier intervention.

There were several limitations to this study. The sample size was small due to the intimate size of ophthalmology residency programs. Additionally, some residents rotating at locations outside of the IRB-covered site were not included. Further studies should strive to include more residents. Further, not every resident performed the same number of slit lamp examinations due to scheduling conflicts and resident location changes outside of IRB coverage. As a result, the mean REBA score could be altered. Further, this study used one grader instead of multiple, which introduces bias into the study. Additionally, correlation analysis was not performed to account for how time of day affects posture, which presents an opportunity for future studies. Lastly, the time spent in each posture was not recorded, which introduces a bias in our study given the activity score of +1 assigned to all slit lamp exams. In the future, recording slit lamp exam length would give a more accurate assessment of posture MSD risk.

Residents were also not masked as to the purpose of the study, introducing a limitation of potential bias from the Hawthorne effect [21]. Due to their awareness of observation, residents may have adjusted their posture that does not normally occur during their clinic practice. Future studies should attempt to mask the purpose in order to reduce potential bias from the Hawthorne effect.

Another notable limitation of this descriptive case study is that the following physical attributes were not obtained from patients and physicians: height, weight, and age. It is important to contextualize these factors in the study of ergonomics, as both patient and physician physical attributes may contribute to varying levels of MSK risk in a real-world setting. This is particularly true for the field of ophthalmology, in which the slit lamp and many surgical instruments are ergonomically best suited for a taller examiner that is often male [10].

Future studies should aim to collect physician personal attributes, such as their height, weight, and age, to help contextualize how patient and physician attributes shape ergonomic risk. Additionally, future studies should examine the indirect examination and ophthalmic surgeries performed by ophthalmology residents to understand the full ergonomic risk ophthalmology residents face.

## Conclusions

In conclusion, ophthalmology residents’ postures demonstrate risk for the development of MSK disorders. Residency programs have an opportunity to improve upon the ergonomic risk factors faced by residents by implementing formal ergonomics training into their curriculum. We are currently conducting an IRB-approved study to ascertain the efficacy of a newly designed, formal ergonomics training program on resident posture to demonstrate the quantitative benefit of resident REBA scores.
